# A new conceptual model for seed germination and seedling tillering of winter wheat in the field

**DOI:** 10.1098/rsos.240723

**Published:** 2025-01-22

**Authors:** Jinping Chen, Peter A. Whalley, Zhongyang Li, Xiaoxian Zhang, Malcolm J. Hawkesford, W. Richard Whalley

**Affiliations:** ^1^ Shangqiu Station of National Field Agroecosystem Experimental Network of China, Shangqiu, Henan Province 476000, People’s Republic of China; ^2^ Seminar for Statistics, Department of Mathematics, ETH Zurich, Zurich, Switzerland; ^3^ Sustainable Soils and Crops, Rothamsted Research, Harpenden AL5 2JQ, UK

**Keywords:** seed germination, seedling tillering, soil water content, air temperature, modelling

## Abstract

Seed germination is a crucial stage in plant development, intricately regulated by various environmental stimuli. Understanding these interactions is essential for optimizing planting and seedling management but remains challenging due to the trade-off effects of environmental factors on the germination process. We proposed a new conceptual model by viewing seed germination as a dynamic process in a physiological dimension, with the influence of environmental factors and seed heterogeneity characterized by a germination speed and a dispersion coefficient. To validate the model, we conducted field experiments by drilling wheat seeds at different dates to establish a temperature gradient and in different plots to create a soil water content gradient. Comparisons with our experimental data and literature results show the model accurately reproduces all germination patterns and the subsequent seedling tillering, with *R*^2^ > 0.95. Our results reveal that within suboptimal temperature range, the seed germination increases asymptotically with temperature, and that as soil water content increases, the germination speed increases initially before decreasing, illustrating the trade-off effect of soil water on bioavailability of water and oxygen. Introducing a physiological dimension enables seed germination and the subsequent tillering process to be modelled as a continuous physiological process, providing deeper insight into plant growth dynamics.

## 1. Introduction

Seed germination is critical not only to plant survival but also to post-germination plant development such as fitness, timing of flowering and dynamics of the plant community [1,2]. Difference between seeds in their response to environmental stimuli is an evolutionary strategy to improve plant survival in natural systems [3]. In managed ecosystems, however, it is desirable for seeds to germinate simultaneously, and germination uniformity is thus a seed quality trait [4–6].

Seed germination is a complex physiological process modulated by environmental cues including light, oxygen (O_2_), temperature and the amount of water available for seeds to imbibe [7]. The onset is water uptake of the quiescent dry seed and the end is the emergence of the embryonic axis out of the seed coat [8]. Several key stages, including solute leakage during imbibition, initiation of metabolic activity for protein synthesis and emergence of the radicles, are distinguishable in germination [9], many of which can be visualized non-invasively through imaging technologies [10,11]. Seed germination has received less attention compared to other topics in plant science [9,12], but the advances in molecular technologies over the past few decades have substantially improved our understanding of the genes and interlocked hormone networks that regulate seed germination [13–15]. The response of gene expression to environmental cues in seed germination has been well documented [16], and network modelling of the transcriptional interactions involved in seed germination has also been constructed to link specific genes and pathways to seed germination and dormancy [17]. Quantitative modelling of seed germination is largely based on the hydrothermal time concept assuming that temperature and water are the main environmental stimuli of seed germination [7,18]. A key feature of the hydrothermal time models is that when temperature and water potential exceed their respective base values (below which seeds remain metabolically inactive), the seed needs to accumulate a sufficient hydrothermal time for its radicle to break the seed coat. Seed heterogeneity is described by viewing the base temperature and/or water potential as random variables [19]. The hydrothermal time model was initially developed for seed germination between the base and the optimal temperatures [7], and has since been generalized to cover a wide range of temperatures as experiments revealed that average germination does not increase linearly with temperature after temperature exceeds a threshold [20,21]. The improved hydrothermal time models include the halo-time model to account for salinity [22] and the oxygen-time model to represent O_2_ stress [23].

Seeds need to absorb sufficient water and thermal energy to germinate. Physically, the linear dependence of hydrothermal time on temperature and water potential, as assumed in the hydrothermal time models, means that the rates at which water and heat flow from soil into the seed are proportional to a water potential gradient and a temperature gradient respectively [7,18,24–26]. While this is true for seed germination in polyethylene glycol on Petri dishes, where the seed–water contact area is constant and the only resistance to seed imbibition is the ability of seed coat to adsorb water, its application to the field could be problematic. Seed imbibition of water from the soil occurs through the seed–water interface, and the imbibing rate depends not only on water potential difference between seed and the water–seed interface, but also on the ability of soil to transport distant water toward the seed and the water–seed interfacial areas to imbibe water, both varying nonlinearly with water potential [27]. Apart from this, temperature fluctuates in the field and the atmospheric O_2_ needs to overcome various resistances before becoming bioavailable to seeds [28,29]. Hydrothermal models use an oxygen time to describe O_2_ sensitivity [23,28,30], but bioavailable O_2_ in the field is not an independent environmental cue; it is controlled by soil water and pore-scale soil structure [31,32], thereby varying with soil texture and soil water content.

This paper aims to propose a new conceptual model to simulate seed germination in the field. We view the metabolic reactions associated with seed germination as a dynamic process in a physiological dimension. The fluctuations in environmental stimuli and seed heterogeneity result in uncertainties in the metabolic reactions, in that physiological advancement that a seed makes at different times and physiological stages varies. We show that such variations can be modelled by a partial differential equation, with the mean of the variations represented by an average germination speed and the variance by a dispersion coefficient. To test the model, we conducted field experiments to measure the germination and seedling development of winter wheat seeds drilled at different dates (to generate a temperature gradient) in plots with different soil water contents (to generate a soil moisture gradient). The novelty of introducing a physiological dimension is that it allows seed germination and subsequent seedling development to be modelled as a continuous physiological process.

The challenge for hydrothermal time models, which require water potential, is that, due to technological limitations, measuring soil water potential higher than 200 kPa in the field using the best available methods is difficult [33,34]. This value is much lower than the optimal soil water potential for wheat seeds. Additionally, *in situ* measurement of dissolved O_2_ is also challenging. This is why most hydrothermal time models were derived from Petri-dish experiments and have limited applications in the field [18,21,35]. This paper aims to bridge this gap.

## 2. Material and methods

### 2.1. The experiment

The field experiments were conducted in the 2018−2019 growing season from October 2018 to February 2019 at Shangqiu Station of the National Agroecological Monitoring Network in China (latitude 34°35′04″ N, longitude 115°34′09″ W). The annual average temperature and precipitation at the experimental site are 13.9°C and 708 mm [36], respectively, with the majority of the rains falling from July to September. Traditional cultivation is maize–wheat rotation with the wheat drilled in October and harvested in the following May. The soil at the site is predominantly fluvio-aquatic, with its bulk density and water-holding capacity (0−30 cm soil) being 1.45 g cm^−3^ and 25% (w/w), respectively. The contents of total N, P and K are 0.62 g kg^−1^, 0.73 g kg^−1^ and 0.845 g kg^−1^, respectively, and those of the associated minerals N, P and K are 37.26 mg kg^−1^, 18.14 mg kg^−1^ and 0.158 g kg^−1^, respectively. The organic matter content and pH of the topsoil are 15.73 g kg^−1^ and 8.83, respectively.

The seedbed was prepared on 2 October 2018 by ploughing the soil to a depth of 10 cm using a conventional rotary tillage after harvesting the maize. Base fertilization was N (150 kg ha^−1^), P_2_O_5_ (75 kg ha^−1^) and K_2_O (150 kg ha^−1^), and their mixture was broadcasted uniformly over the surface before ploughing them into the soil. Winter wheat variety Dwarf 985, a cultivar widely grown by local farmers, was used as the model plant. Wheat seeds at a planting density of 435 plants m^−2^ were drilled approximately to the depth of 5 cm on 5 October 2018, 10 October 2018 and 15 October 2018, with the soil water content maintained at 75% of the field capacity. Added to each drilling date were three soil water treatments by maintaining the soil water content not less than 75, 65 and 55% of the field capacity, respectively. Every treatment had three replicas and they were organized randomly in 27 plots, each measured 6 × 5 = 30 m^2^. To avoid lateral water flow, there was a 50 cm buffer strip between adjacent plots. A combined soil moisture and temperature sensor probe (TRIME -BT) was inserted to a depth of 100 cm at the centre of each plot to measure soil water content and temperature simultaneously at depths from 5 to 100 cm with varying intervals (10 cm in the topsoil and 20 cm in the subsoil) using a data logger (Em50, Decagon Device Inc., USA). Whenever the sensor-measured soil water content in the controlled zone (0−20 cm in germination stage and 0−40 cm in seedling tillering stage) was below the designed soil water content (55, 65 and 75% of the field capacity), we topped up the soil water to 85% of the field capacity with the irrigation amount for each treatment calculated based on the measured soil water content in each plot and 85% of the field capacity.

Seedling emergence and germination were measured daily on each plot; when the seedlings started to tiller, we counted the tiller numbers from daily to weekly, in a 1 m long section randomly selected from a row in each plot.

### 2.2. The model

Seed germination is a physiological process involving various metabolic reactions. From seed imbibition to emergence of the radicle out of the seed coat, there are several distinguishable reactions. These reactions can be viewed as an irreversible moving event in a physiological dimension. We represent this physiological dimension by *x* and time by *t*, with *x* = 0 representing the onset of water imbibition of the quiescent dry seed and *L*_0_ representing the stage of seedling emergence. The time step is Δ*t*, and the frequency probability distribution that the seed makes an *r* (*r *> 0) physiological advancement in one time step is *p*(*r*). If the seed is at stage *x* = 0 initially (i.e. *t* = 0), and the probability that the seed moves to stage *x* after *m* time steps is $P_{m}(x)$, $P_{m}(x)$ can be calculated from


(2.1) $$P_{m}\left(x\right)=\int_{0}^{x}p\left(r\right)P_{m-1}\left(x-r\right)\;dr.$$

We Taylor-expand $P_{m-1}(x-r)$ as follows:


(2.2) $$P_{m-1}\left(x-r\right)\approx P_{m-1}\left(x\right)-r\frac{\partial P_{m-1}}{\partial x}+\frac{r^{2}}{2}\frac{\partial^{2}P_{m-1}}{\partial x^{2}}.$$

Substituting equation (2.2) into equation (2.1) yields


(2.3) $$P_{m}\left(x\right)=P_{m-1}\left(x\right)-\left(\int_{0}^{x}rp\left(r\right)\;dr\right)\frac{\partial P_{m-1}}{\partial x}+\frac{1}{2}\left(\int_{0}^{x}r^{2}p\left(r\right)\;dr\right)\frac{\partial^{2}P_{m-1}}{\partial x^{2}}.$$

In the limit Δt→0, equation (2.3) reduces to


(2.4) $$\frac{\partial \rho }{\partial t}=D_{G}\frac{\partial^{2}\rho }{\partial x^{2}}-u_{G}\frac{\partial \rho }{\partial x},$$

where $u_{G}=\lim_{\Delta t\to 0}\int_{0}^{x}rp\left(r\right)\;dr/\Delta t$ is average germination speed, $D_{G}=\lim_{\Delta t\to 0}0.5\int_{0}^{x}r^{2}p\left(r\right)\;dr/\Delta t$ is germination dispersion coefficient, and $\rho =P_{m}(x)$. Following the Kramers–Moyal expansion [37,38], because of the dependence of *p*(*r*) on Δ*t*, *u_G_* and *D_G_* are finite when Δt→0. The expansion is up to second order, and *u*_*G*_ and *D*_*G*_ are proportional to the first and second moments of *p*(*r*) respectively. If there are *N*_0_ seeds at stage *x* = 0 initially (i.e. *t* = 0), the equation for their germination and the associated initial and boundary conditions are


(2.5) $$\begin{array}{l} \frac{\partial n}{\partial t}=D_{G}\frac{\partial^{2}n}{\partial x^{2}}-u_{G}\frac{\partial n}{\partial x},\\ n\left(x,0\right)=0;\\ n\left(0,t\right)=\left\{ \begin{array}{l} N_{0},\;t=0\\ 0,\;\;\;\;t>0 \end{array}\right.\\ \frac{\partial n}{\partial x}{|}_{x=L_{0}}=0, \end{array}$$

where $n=\rho N_{0}$.

The analytical solution of equation (2.5) is [39]


(2.6) $$n\left(x,t\right)=N_{0}\sqrt{\frac{1}{\pi \tau }}\exp\left(\frac{\alpha \left(X-\tau \right)}{4\tau }\right)-\frac{1}{2p}\exp\left(\frac{X}{\alpha }\right)erfc\left(\frac{X+\tau }{4\tau \alpha }\right),$$

where $\alpha =D_{g}u_{g}^{-1}L_{0}^{-1}$, $\tau =u_{g}tL_{0}^{-1}$, $X=xL_{0}^{-1}$. When $u_{g}L_{0}D_{g}^{-1}\gg 1$, the second term in equation (2.6) is negligible compared to the first one.

Compared with traditional hydrothermal models and their variants [18,21,30,40], the novelty of introducing a physiological dimension is that it allows seed germination and the subsequent tillering process to be modelled as a continuous physiological process. If each radicle emerging out of the seed coat has the potential to develop into *γ* tillers and we use $x\in (L_{0},\;L_{1})$ to represent the physiological range of the tillering process, from the same principle, we can derive the following equation for the tillering process:


(2.7) $$\frac{\partial N}{\partial t}=D_{T}\frac{\partial^{2}N}{\partial x^{2}}-u_{T}\frac{\partial N}{\partial x},$$

where *u*_*T*_ and *D*_*T*_ are averaging tillering speed and tillering dispersion respectively and *N* is the number of potential tillers at stage *x* and time *t*. Equation (2.7) for tillering process is linked to equation (2.5) for seed germination at *x* = *L*_0_ as follows:


(2.8) $${\left(-D_{T}\frac{\partial N}{\partial x}+u_{T}N\right)|}_{x=L_{0}^{+}}=\gamma \cdot u_{G}\cdot n{|}_{x=L_{0}^{-}}$$

to ensure that the end of the germination is the onset of the tillering development, and that, on average, each seedling has the potential to develop into *γ* tillers. Germination can be calculated analytically using equation (2.6), but its combination with tillering, or when *u*_*G*_ and *D*_*G*_ vary with time, needs to be solved numerically. To avoid numerical dispersion and instability, we solve equations (2.5) and (2.7) collectively using the following second-order finite-volume method:


(2.9) $$m_{x_{i}}^{t_{j}+\delta t}=m_{x_{i}}^{t_{j}}+\frac{\delta t}{\delta x}{\left(q_{w}-q_{e}\right)}^{t_{j}},$$

where *δt* and *δx* represent time step and physiological step, respectively, satisfying δx>u⋅δt and uΔx/D<2 [41,42], and other terms are


(2.10) $$\begin{array}{rl} & q_{w}=\left\{ \begin{array}{ll} 0 & x_{i}=0\\ \gamma \cdot um_{x_{i}-\delta x} & x_{i}=L_{0}+\delta x\\ D\left(m_{x_{i}-\delta x}-m_{x_{i}}\right)/\delta x+u\left(m_{x_{i}-\delta x}+m_{x_{i}}\right)/2 & \text{otherwise}, \end{array}\right.\\ & q_{e}=\left\{ \begin{array}{ll} um_{x_{i}} & x_{i}=L_{0},L_{1}\\ D\left(m_{x_{i}}-m_{x_{i}+\delta x}\right)/\delta x+u\left(m_{x_{i}}+m_{x_{i}+\delta x}\right)/2 & \text{otherwise}, \end{array}\right. \end{array}$$

where *m* = *n*, *D* = *D*_*G*_ and *u* = *u*_*G*_ for germination in $x\in (0,\;L_{0})$, and *m* = *N*, *D* = *D*_*T*_ and *u* = *u*_*T*_ for tillering in $x\in (L_{0},\;L_{1})$. In all simulations, we view the process from the onset of seed imbibition to seedling emergence as one physiological unit defined by *L*_0_ = 1, and the subsequent tillering process as another physiological unit defined by *L*_1_ = 2.

### 2.3. Model testing

The model is derived for viable seeds that germinate. In the field, there are seed losses and seed dormancy, but it is impossible to estimate such seed losses in large-scale field experiments. Since the number of seeds drilled in different treatments was approximately the same, as an approximation, the number of viable seeds in each treatment was estimated as a relative value, obtained by normalizing the emergent seedlings by the number of viable seeds in the treatment that had the most emergent seedlings. Each treatment has three replicates, and in modelling we pooled their results. Each emergent seedling has the potential to develop into *γ* tillers, which was treated as a known parameter, estimated based on the number of emergent seedlings and the numbers of tillers at the end of the experiment.

Seed germination is represented by *u*_*G*_ and *D*_*G*_, and the tillering by *u*_*T*_ and *D*_*T*_. Since germination is independent of tillering, we estimated *u*_*G*_ and *D*_*G*_ first. For each treatment, we selected a series of *u*_*G*_ values and a series of *D*_*G*_ values. For each pair of *u*_*G*_ and *D*_*G*_, we simulated a germination pattern and then calculated its coefficient of determination (*R*^2^) with the measured data. The values of *u*_*G*_ and *D*_*G*_ for each treatment were those that gave the highest *R*^2^. Once *u*_*G*_ and *D*_*G*_ were determined, we extended the simulation to the tillering development and estimated *u*_*T*_ and *D*_*T*_ using the same method. The values of *u*_G_, *D*_*G*_, *u*_*T*_ and *D*_*T*_ were constant for each treatment, but they varied among treatments due to the difference in their soil moisture content and planting date. All calculations and curve fittings were executed using MATLAB (Version: 9.13.0 (R2022b), 2022, The MathWorks Inc., Natick, MA).

#### 2.3.1. Model validation

To validate if the model and the values of the germination coefficients estimated from the 2018 experiments can be used for prediction, we applied them to calculate the seed germination patterns of the same wheat cultivar measured in 2011 and 2013 on the same site. In 2011, the seeds were drilled on 21 October to the soil with different water contents, while in 2013, they were drilled from 15 October to 25 October (effectively generating a temperature gradient) to the soil with the soil water content kept at 65% of the field capacity.

#### 2.3.2. Comparison with hydrothermal time model

Soil water potential is difficult to measure in the field due to technological limitations, particularly for soil water potential in the range that is optimal for wheat seed germination. We thus compared the proposed model and the hydrothermal time model for reproducing the germination patterns of durum wheat seeds measured in laboratory under different water potentials in a loamy soil and a clay soil [43]. The comparison was quantified using the *R*^2^ value of each model for reproducing the experimental data. The soil water potential in different treatments was controlled by pressure chambers. A detailed description of the experimental procedure is given in Rinaldi *et al.* [43]. The base water potential in the hydrothermal time model was described by the Weibull function [44].

## 3. Results

### 3.1. Model calibration

**Figure 1.**
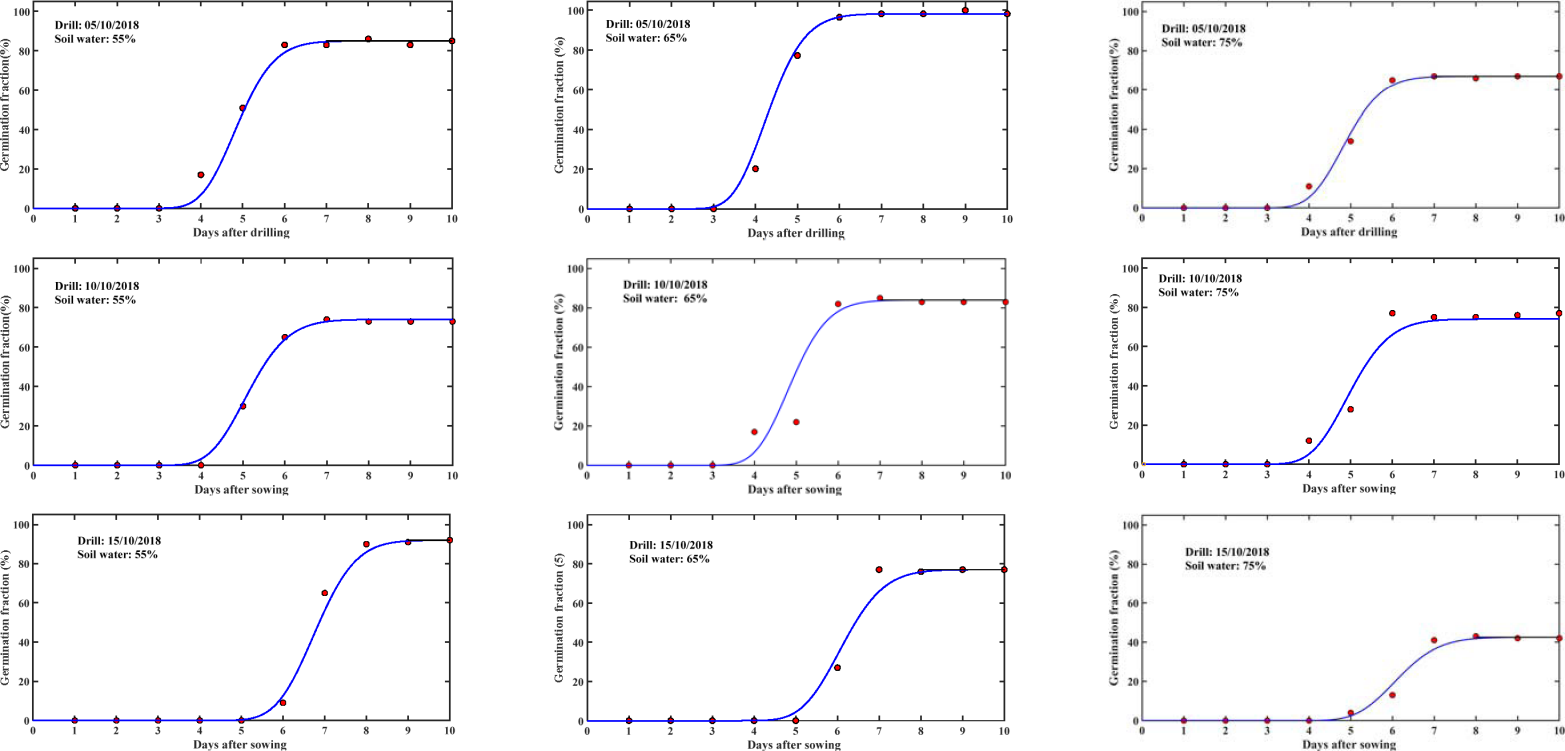
Measured (symbols) and simulated (solid lines) percentage of germinated seeds drilled at different dates in plots under different soil water contents (expressed as % of the field capacity) as shown in the legends.

**Figure 2.**
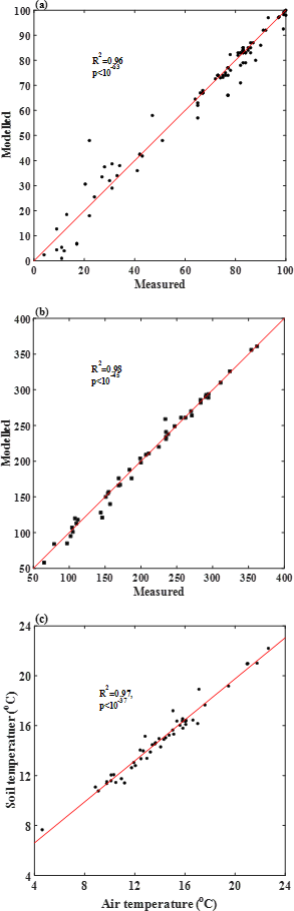
Scatterplots comparing the measured and modelled percentages of germinated seeds (*a*), and the simulated and measured numbers of tillers (*b*). Correlation between air temperature and soil temperature at a depth of 5 cm (*c*).

We first calibrated the model using the germination data measured in 2018 to obtain the model parameters. Figure 1 compares the simulated germination patterns with the experimental data for all treatments. The experiment was conducted in natural conditions without protection from wildlife and the number of seedlings was counted daily. In some treatments, there was a slight decrease in seedlings, likely to be caused by bird predation. However, overall, the modelled and measured results agree well, with *R*^2^ > 0.95, indicating that the model captures the physiological development involved in the seed germination. Figure 2*a* shows the statistics between the measured and simulated results for all treatments.

The results in figure 1 were calibrated by treating the average germination speed and germination dispersion coefficient as constants for each treatment. However, the values of the two germination parameters vary with treatments, because of the difference in their soil water content and temperature. Seed germination is controlled by both temperature and bioavailable water in the vicinity of the seeds. We measured soil temperature at different soil depths but considering that the model was developed for practical application in the field, we used air temperature measured in a weather station on the experimental site to analyse the impact of temperature and soil moisture on the two germination parameters. This approach was reasonable as soil temperature at a depth of 5 cm, where the seeds were drilled to, was closely related to the air temperature measured in the weather station (figure 2*c*).

**Figure 3.**
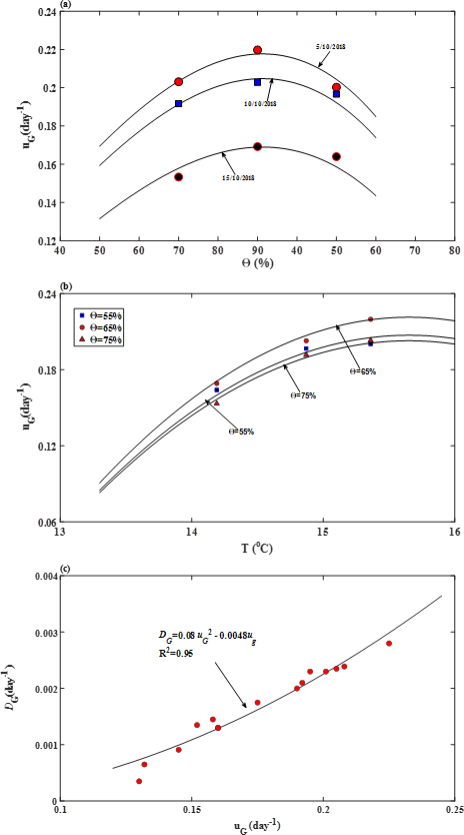
(*a*) Change in average germination speed with soil water content (expressed as % of the field capacity) for seeds drilled at different dates (shown in the panel). (*b*) Change in average germination speed with air temperature for different soil water content. (*c*) The relationship between average germination speed (*u*_*G*_) and dispersion germination coefficient (*D*_*G*_) that characterize the heterogeneity of seeds in their response to changes in soil water and temperature.

Temperature and soil moisture fluctuated when the seeds were germinating. For each treatment, we used the time-averages of temperature and soil water to describe their effects. Figure 3*a*,*b* shows the changes in average germination speed with soil moisture and temperature, respectively, and figure 3*c* shows the relationship between the germination dispersion coefficient and the average germination speed. They are closely correlated, allowing the latter to be estimated from the former.

### 3.2. Verification

**Figure 4.**
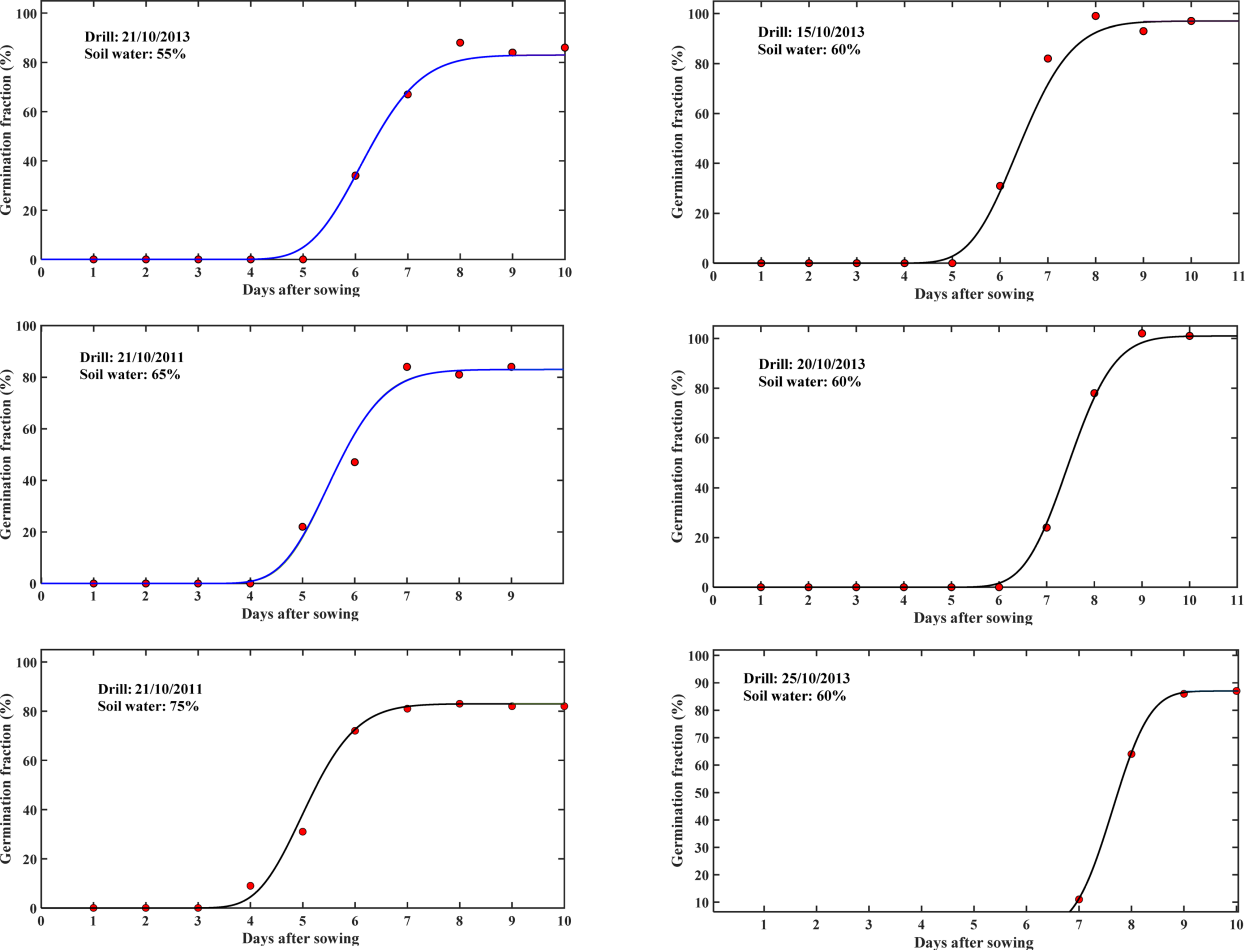
Comparison of the simulated germination patterns (solid lines) using the model and *u*_*G*_ and *D*_*G*_ estimated from figure 3 and those measured (symbols) in 2011 (left column) for seeds drilled at the same date but under different soil water contents (marked in the panels), and in 2013 (right column) for seeds drilled at different dates but at the same soil water content (shown in the panels).

The changes in average germination rate and germination dispersion coefficient with soil water content and temperature shown in figure 3 can be used to predict seed germination under different combinations of temperature and soil moisture. We verify this using the seed germination patterns of the same wheat cultivars measured in 2011 and 2013 under different soil moisture and planting date (i.e. different temperatures) on the same site. Again, soil moisture and air temperature in the model for each treatment were estimated as time-averages over the germination period, and the values of the associated average germination speed and germination dispersion coefficient were estimated from the smooth curves in figure 3. Figure 4 compares the measured and predicted germination patterns for each treatment. The scatterplot in figure 2*a* shows the statistics between the measured and predicted germination patterns. Overall, they agree well with *R*^2^ > 0.95.

### 3.3. Comparison with hydrothermal time model

**Figure 5.**
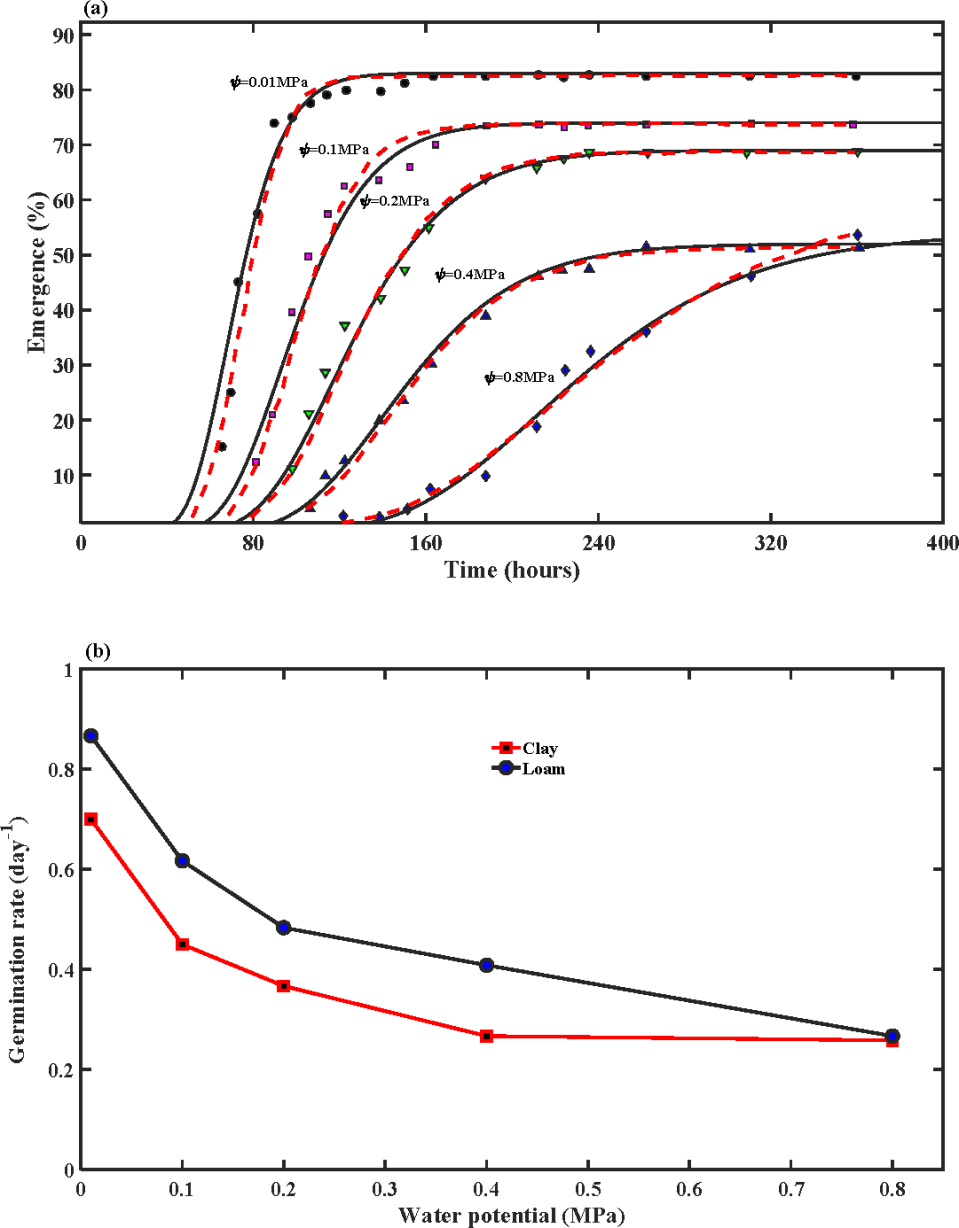
(*a*) Comparison of the simulated seedling emergence patterns using the proposed model (the solid black lines) and the hydrothermal time model (the red dashed lines) with those measured from the loamy soil under different water potential (the symbols). (*b*) The average germination speed not only decreases nonlinearly as soil water potential increases but also varies with soil texture.

Figure 5*a* compares our model and the hydrothermal time model against the measured germination patterns of wheat seeds under different soil water potential in the loamy soil; the results for the clay soil are similar (not presented). For each soil water potential, the average germination speed and the germination dispersion coefficient were constant and calculated by calibrating the modelled results to the measured data. Visually, there is a slight difference between the two models in reproducing the measured data (figure 5*a*), but in terms of *R*^2^, there is no difference between them, both giving *R*^2^ > 0.96; figure 5*b* shows the variation of the average germination speed with soil water potential for the two soils.

### 3.4. Extending to tillering

**Figure 6.**
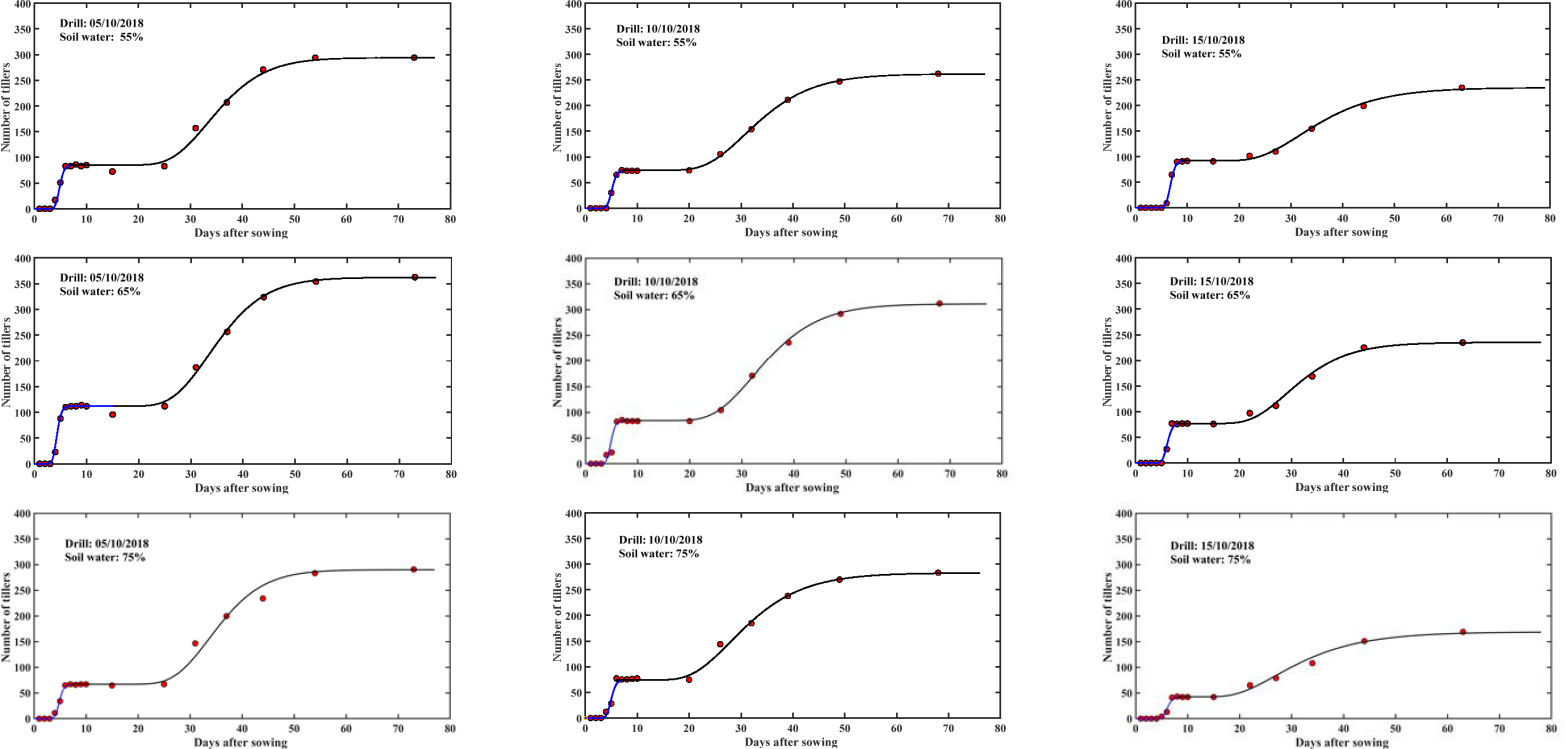
Combining seed germination and the subsequent tillering as a continued process, we compared the changes in measured (symbols) and simulated (solid lines) tiller numbers per metre with time for seeds drilled at different dates in plots under different soil water contents (shown in the legends).

**Figure 7.**
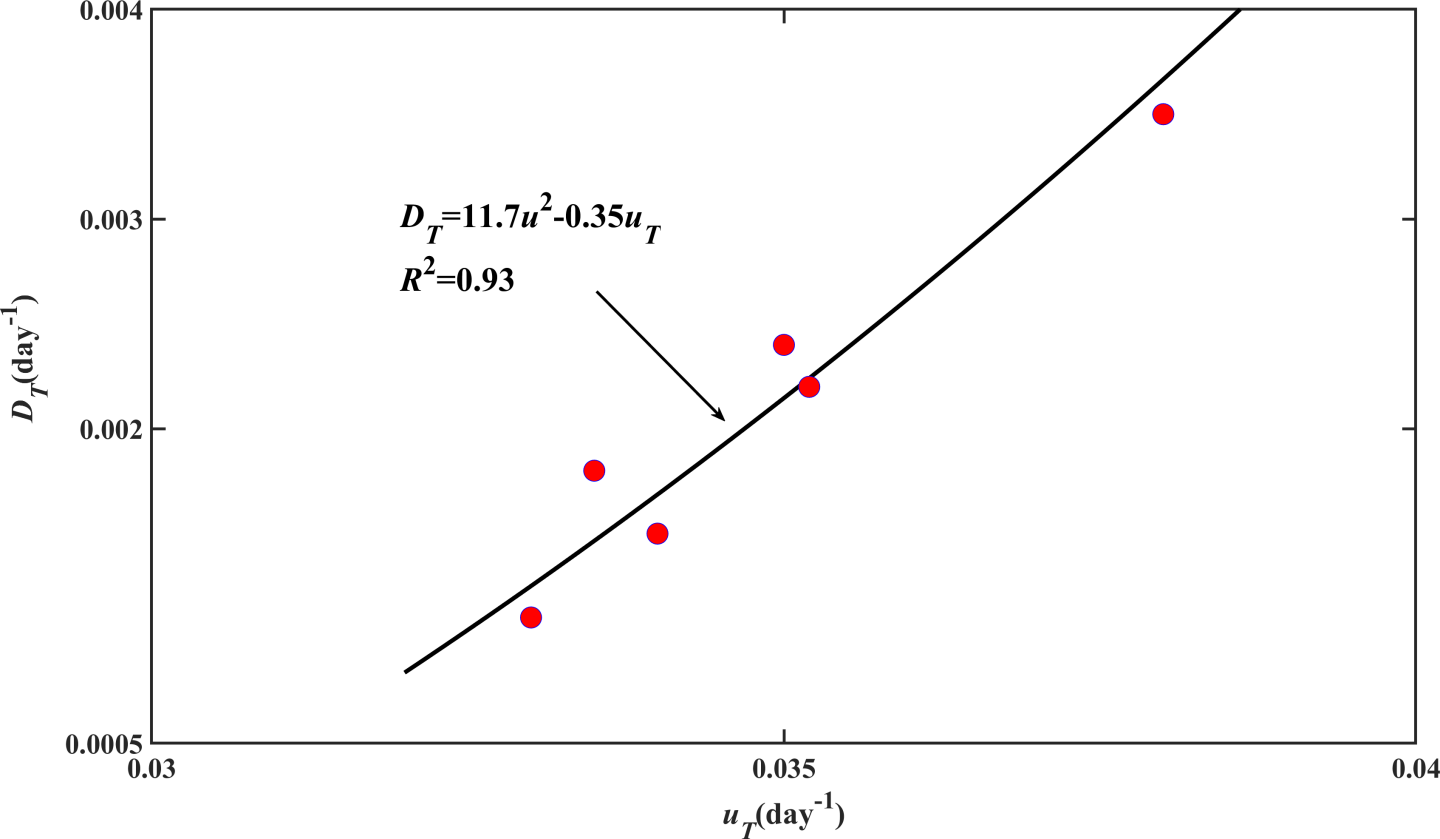
The relationship between the average tillering speed (*u*_*T*_) and its associated tillering dispersion coefficient (*D*_*T*_) that characterize the heterogeneity of seedlings in their response to environmental changes.

Each newly emerged seedling at time *t* has the potential to develop into *γ* tillers. The tillering development process was modelled by equations (2.9) and (2.10). The average tillering speed *u*_*T*_ and tillering dispersion coefficient *D*_*T*_ were constant for each treatment, but they varied with treatments. Figure 6 compares the simulated and measured tillering patterns. The scatterplot in figure 2*b* shows the statistics between the simulated and measured tillers. Overall, they agree well with *R*^2^ > 0.95. Figure 7 shows that the average tillering speed and the tillering dispersion coefficient are correlated, and the latter can be estimated from the former.

## 4. Discussion

Soil environments regulate seed germination and the subsequent seedling development and establishment. In agricultural management, the ability to predict them using easy-to-measure or forecastable environmental stimuli is highly desirable. For seed germination, the environmental stimuli are temperature, soil moisture and dissolved O_2_. Our model is developed based on this principle by using soil moisture and air temperature as the environmental cues. Our focus is seed germination, but the model can combine seed germination and seedling tillering as a continuous physiological process (figures 3 and 7).

### 4.1. Effect of soil water content

**Figure 8.**
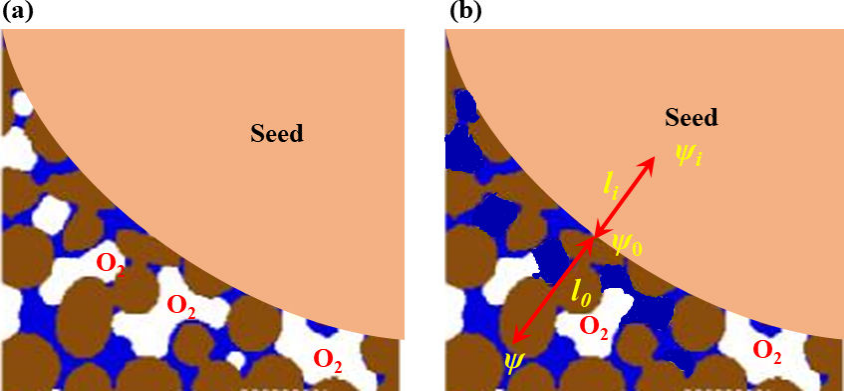
Schematic showing the impact of soil water and soil structure on seed germination. Brown areas represent soil particles, blue represents water and white represents water–air interface. The seed absorbs water and oxygen (O_2_) through its contact area with water. Oxygen dissolves at the water–air interface and diffuses through the water to reach the seed–water interface, where it becomes bioavailable to the seed. As soil water content changes from condition (*a*) to condition (*b*), the water–air interfacial area decreases, affecting bioavailable O_2_ due to the reduced area for oxygen to dissolve and increased distance for dissolved oxygen to move from the water–air interface to the seed.

We use soil water content instead of soil water potential not only because the former is easy to measure and forecastable [45,46], but also because it can calculate water flow from soil into seed [21]. Physically, water flow from soil into seed is driven by a water potential difference between them. The hydrothermal time models do not directly calculate water flow, but it implicitly assumes that the time it takes a seed to germinate is proportional to the water that flows into the seed. Mathematically, the hydrothermal model calculates seed germination assuming that the germination rate of a fraction of seed lot, *t*_*g*_, is proportional to the difference between water potential (*ψ*) in the medium and a reference water potential (*ψ*_*i*_) as $1/t_{g}=(\psi -\psi_{i})/H_{g}$, where *H*_*g*_ is the hydrothermal time assumed to be constant. Hydraulically, this is Darcy’s law describing water flow in porous materials including soils and seeds, where *H_g_* is called hydraulic resistance in soil physics and hydrology [47]. Instead of using water potential, we used soil water content to describe water flow from the soil into the seeds, which is physically the same as water flow implicitly described in the hydrothermal model. To demonstrate this, figure 8 illustratively shows a seed and its surrounding soil that is partly filled with water. If water potential inside the seed, on the seed–soil interface and in the soil are represented by *ψ*_*i*_, *ψ*_0_ and *ψ*, respectively, water flow from the soil to the seed–water interface is $q_{m}=A(\psi )k_{m}(\psi )\left[\psi -\psi_{0}\right]/l_{0}$, and water flow from the seed–water interface into the seed is $q_{s}=A(\psi )k_{s}\left[\psi_{0}-\psi_{i}\right]/l_{i}$, where *A*(*ψ*) is the seed–water interfacial area, and $k_{m}(\psi )$ and $k_{s}$ are the hydraulic conductivity of the soil and seed respectively; mass balance requires *q*_*m*_ = *q*_*s*_. For seeds germinating in polyethylene glycol, *A*(*ψ*) = *A*_0_ is a constant and $k_{m}\left(\psi \right)\gg k_{s}$. Therefore, $\psi \approx \psi_{0}$, and the seed imbibition rate is $q_{s}=A_{0}k_{s}(\psi )(\psi -\psi_{i})$. Compared to hydeothermal time model, this is equivalent to $1/t_{g}\propto q_{s}=A_{0}k_{s}\left(\psi \right)\left(\psi -\psi_{i}\right)$, with the hydrothermal time $H_{g}=1/A_{0}k_{s}(\psi )$ being the hydraulic resistance.

The hydrothermal time model assumes the hydraulic conductivity of all seeds is the same and independent of water potential. Instead, it models seed heterogeneity and their response to environmental changes by assuming that water potential inside the seed (*ψ*_*i*_) is a random number varying not only among seeds but also with soil water potential. This approach can reproduce the germination patterns under different soil water potentials (figure 5*a*), but there is no experimental evidence to support these assumptions. In the field, seed–water interfacial area and soil hydraulic conductivity both decrease nonlinearly as soil water potential increases [27]. Therefore, with an increase in water potential, water flow rate from the soil into the seeds decreases. This is the physical mechanism underlying the nonlinear decrease in average germination speed as the soil water potential increases (figure 5*b*).

In plant–soil systems, water flow in soil can be described using either soil water potential gradient or soil water content gradient as the driving force [48,49]. Referring to figure 8, if soil water contents associated with water potential in the soil (*ψ*) and water potential on the seed–soil interface (*ψ*_0_) are represented by *Θ* and *Θ*_0_, respectively, water flow from the soil to the water–seed interface can be rewritten as $q_{m}=k_{m}(\Theta )\left\{\left[\psi (\Theta )-\psi_{0}\right]{\left(\Theta -\Theta_{0}\right)}^{-1}\right\}A(\Theta )(\Theta -\Theta_{0})/l_{0}$, where $k_{m}(\Theta )\left\{\left[\psi (\Theta )-\psi_{0}\right]{\left(\Theta -\Theta_{0}\right)}^{-1}\right\}=D_{m}(\Theta )$ is called soil diffusivity in soil physics [48]. Therefore, water flow from soil into the seed is $q_{m}=D_{m}(\Theta )A(\Theta )(\Theta -\Theta_{r})/l_{0}$; this is an approach widely used for modelling water flow in soil–plant systems [49]. Germination speed is proportional to seed imbibition rate, and we hence have $u_{G}\propto D_{m}(\Theta )A(\Theta )(\Theta -\Theta_{0})/l_{0}$. Since $D_{m}(\Theta )$ and A(Θ) both increase with soil water content, the germination speed should increase with soil water content monotonically if soil water is the only limiting factor. This is the case for seed germination in the pressure chambers (figure 5*b*), but not in the field (figure 3*a*) where the average germination speed decreased when soil water content was increased from 65 to 75% of the field capacity. This indicates the presence of other factors which played a role in the moisture effect on seed germination. This factor is oxygen (O_2_).

### 4.2. Combined effects of temperature, soil water and bioavailable O_2_

Dissolved O_2_ in the proximity of seed is another environmental cue essential for respiration in seed germination in the field [50]. The sensitivity of seed germination to O_2_ has been well documented [50]; however, unlike indoor experiments where O_2_ supply was controlled [30], dissolved O_2_ in the proximity of seeds in the field emanates from the atmosphere. For atmospheric O_2_ to become bioavailable, it needs to move into the soil, dissolve at the water–air interface and then move in water constrained in the pore space to where the seeds are [51]. As a result, bioavailable O_2_ to seeds in soil is not an independent cue but varies with soil water content [52,53]. Referring to figure 8, as soil water in an initially dry soil increases, the water–air interfacial area and seed–water contact area for O_2_ dissolution and seed adsorption both increase. As a result, the germination speed increases with increasing soil water content (figure 3*a*). However, when soil water content exceeds a threshold, a further increase reduces the available water–air interface for O_2_ to dissolve and increases the distance between the water–seed interface and soil–seed interface for dissolved O_2_ to move. That is, bioavailable O_2_ to seeds varies with soil water content in a ‘hump shape’, peaking at an intermediate soil water content. For seeds, this means that with the increase in soil water content, their germination speed increases initially before plateauing or declining after bioavailable O_2_ becomes the limiting factor (figure 3*a*). This is consistent with experimental findings [30], but differs from that in figure 5*b* which does not show a noticeable O_2_ stress.

Bioavailable O_2_ in the field is influenced by a multitude of factors [54]. The difference between the field experiment shown in figure 3 and the chamber experiment shown in figure 5*b* is that, apart from different cultivars, seeds in the latter were covered by a 2.5 cm soil layer with a bulk density of 1.1 g cm^−3^, while in the field experiment, the seeds were drilled to a depth of 5 cm and the bulk soil density was 1.45 g cm^−3^. The density of soil particles is approximately 2.67 g cm^−3^, and the porosity of the thin soil layer in the chambers was 58.8%, compared to 45.6% for the soil in the field. The ability of soil to diffuse gaseous O_2_ increases with soil porosity in a power-law function with an exponent of approximately 10/3 [55–57]. The ability of the thin soil layer to transport O_2_ is more than two times that of the field soil.

The combined impact of water flow from soil into seeds and O_2_ stress can be collectively described by an approach used for modelling moisture sensitivity of soil respiration [31,58,59]: $u_{G}\propto \Theta^{\mu }{\left(\Theta_{0}-\Theta \right)}^{\kappa }$, where *Θ*^*µ*^ represents that water flow from soil to seeds increases with soil water content, while $(\Theta_{0}-\Theta {)}^{\kappa }$, where *Θ*_0_ is a critical soil water content, represents that when soil water content exceeds a threshold, O_2_ stress occurs, reducing seed germination.

**Figure 9.**
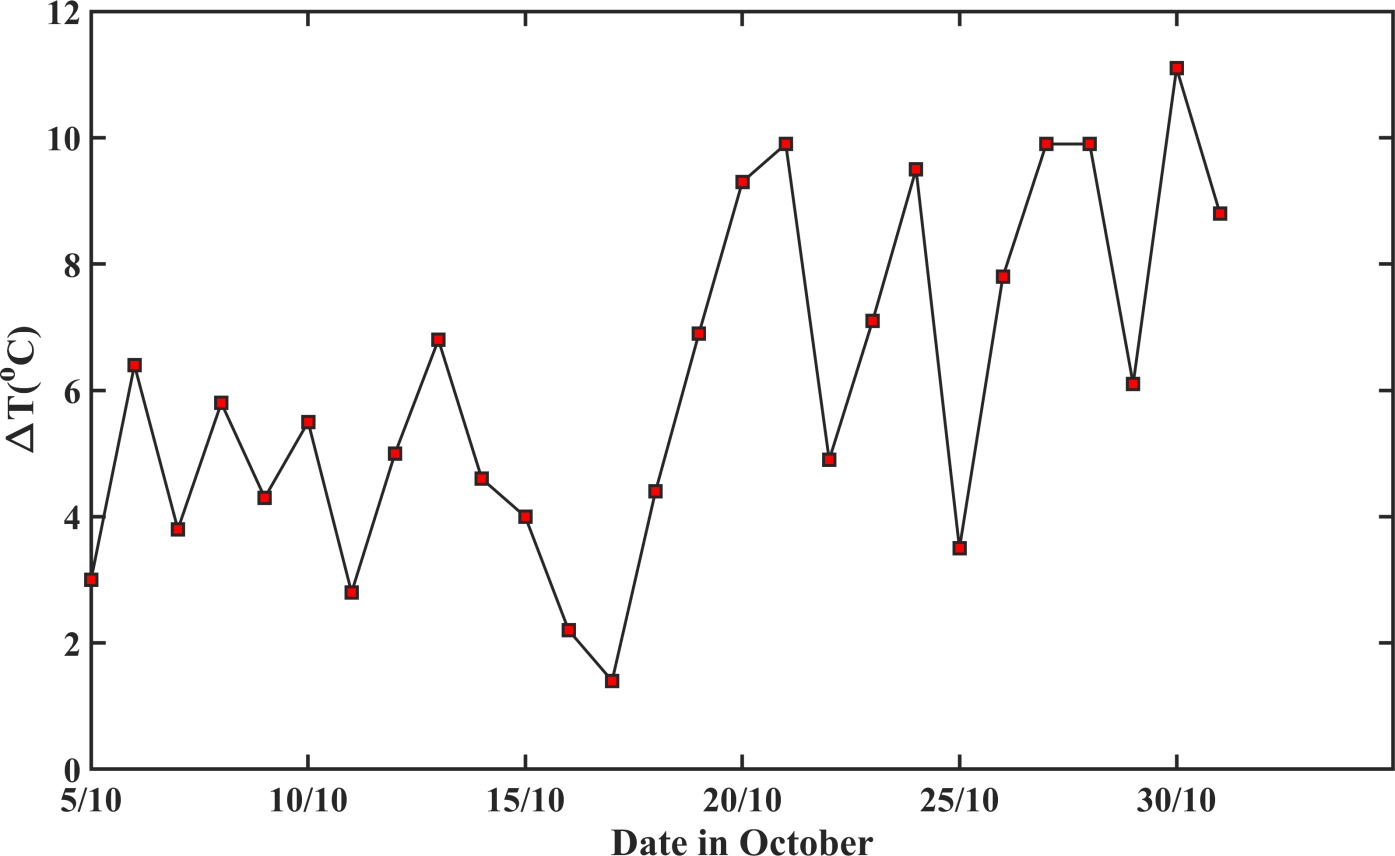
The change in diurnal temperature amplitude (Δ*T*), after the seeds were first drilled on 5 October 2018.

The early hydrothermal time models were derived from controlled Petri-dish experiments assuming average germination rate increases with temperature linearly in the suboptimal range [60]. This, however, does not apply to the field (figure 3*b*), where the soil environments are complicated and temperature fluctuates. The highest temperature in our experiment was 24.6°C on the 8 October and subsequently, the temperature was below 20°C. Therefore, supra-optimal temperature was unlikely the cause of the nonlinear increase in germination rate with temperature, and factors unique in the field are likely the reason. Studies over the past few decades have shown abscisic acid (ABA) and gibberellin (GA) are the hormonal signals linking environmental cues to gene expression in seed germination, and their balance is a key factor regulating seed dormancy and germination [13,30]. Alternating temperature, such as the diurnal temperature change in the field, has been found to increase the GA/ABA ratio by suppressing ABA synthesis, thereby enhancing germination and reducing dormancy [61,62]. Figure 9 shows the amplitude of the diurnal temperature fluctuation, which could be a reason why the average germination speed nonlinearly increases with temperature. This is corroborated by other findings that fitting the time course of seed germinations under alternate temperature using the hydrothermal time model needs to increase the base temperature as diurnal temperature amplitude increases [29]. It is worth noting that the nonlinear increase in germination speed with temperature also varies with soil water content (figure 3*b*), in that the increasing rate was faster when the soil water content was at 65% of the field capacity than at 55 and 75% of the field capacity. When soil water content (hence bioavailable O_2_) is the same, the temperature effect on the average germination speed can be described by $u_{G}\propto a\left(T-T_{0}\right)-b{\left(T-T_{0}\right)}^{2}$, where *T*_0_ is a critical temperature below which seeds become dormant, and *a* and *b* are parameters.

The diffusivity of soil for water flow increases with soil water content in a power-law function [31], i.e. $D_{m}(\Theta )A(\Theta )\propto \Theta^{\beta }$. Since bioavailable O_2_ for seeds in soil depends on soil water content, the combined effect of temperature, soil water and O_2_ on average germination speed can be collectively described by


(4.1) $$u_{G}=\left[a\left(T-T_{0}\right)-b{\left(T-T_{0}\right)}^{2}\right]\left[\Theta^{\beta }\left(\Theta -\Theta_{r}\right)\right]{\left(\Theta_{0}-\Theta \right)}^{\beta }.$$

Representing soil water content as a fraction of the field capacity, the values of the parameters in equation (4.1) in our experiments are *a* = 1.42 (day^−1^/*T*), *T*_0_ = 12.6°C, *b* = 0.23 day^−1^/*T*^2^, *β* = 1.2, *ϴ*_0_ = 1.0 and *ϴ*_*r* _= 0.05. Figure 3 compares the germination speed calculated from equation (4.1) with those directly estimated for each treatment.

### 4.3. From seedling to tillering

Studies of tillering modelling are limited despite its close correlation with grain yield [63,64]. This is probably because, in addition to water and temperature, tillering also depends on other factors such as nutrients. The focus of this paper is on seed germination, but introducing a physiological dimension enables us to combine seed germination and tillering as a continuous physiological process. The effects of biotic and abiotic factors on the tillering were collectively described by an average tillering speed and a tillering dispersion coefficient. The close agreement between the simulated and measured tillering process indicates that the model captured the key mechanisms underlying the tillering development. Since soil nutrients were not measured, we were unable to separate the impact of nutrients from other environmental factors in the same way as we did for the seed germination. The number of tillers into which each emergent seedling can develop was estimated based on experimental data, but it is a genetic parameter and could be estimated from the genome-scale metabolic network model, though achieving this requires further work. Notwithstanding this, modelling seed germination and the subsequent tillering as a single process is a step forward.

### 4.4. Comparison with hydrothermal time model

The proposed model simulates seed germination as a dynamic event in a physiological dimension, differing conceptually from other models. The hydrothermal time models were developed from Petri-dish experiments using polyethylene glycol to adjust water potential, where the solution is well oxygenated and seed imbibition is proportional to a water potential difference [24]. This differs from seeds in the field where seed–water contact area for water to flow from soil into the seed and hydraulic conductivity of the soil both vary with soil water potential (figure 8). Therefore, seed imbibition and the associated germination speed in soil vary with water potential nonlinearly, rather than linearly as observed in the Petri-dish experiments [24]. This is evident in figure 5*b* where the average germination speed decreases nonlinearly as soil water potential increases. Also, soil hydraulic conductivity depends on soil texture and, as a result, the relationship between germination speed and soil water potential varies with soil types (figure 5*b*). For example, the loamy soil in figure 5*b* is more hydraulically conductive than the clay soil under the same water potential [27], and seed germination in the former was hence faster than in the latter (figure 5*b*).

The hydrothermal time model describes O_2_ sensitivity using an oxygen-time [23,30]. While this is valid for seed germination in polyethylene glycol where water potential and oxygen were made artificially independent of each other [65], bioavailable dissolved O_2_ in the field is not an independent environmental cue, but controlled by soil water content (figure 8). In the field, the atmospheric O_2_ needs to move into soil, dissolve at the water–air interface and then move to the seed–water interface prior to being imbibed by the seeds (figure 8). Since O_2_ dissolution and its subsequent movement are modulated by soil water, bioavailable dissolved O_2_ to seeds in the field is not an independent stimulus as assumed in the oxygen-time model [30,50,65].

The hydrothermal time models and their variants work equally well as the proposed model if soil water potential and bioavailable O_2_ can be measured accurately (figure 5). However, because of technological limitations, there are no methods that can reliably measure soil water potential in the range that is optimal for seed germination in the field [33]. This explains why most seed germination models were derived from laboratory experiments with limited applications in the field. The model we proposed overcomes this limitation.

Practical application of the germination model is to predict how planting date and soil management practice affect seed germination and the subsequent seedling development, using forecastable environment factors. Air temperature and topsoil water content can now be forecasted weeks in advance. We hence used them to estimate the average germination speed and dispersion coefficient to model seed germination and subsequent tillering development. Tests against historical data measured from the same site demonstrated that these methods can be used for prediction (figure 4).

It is impossible to count the viable seeds in the field, and their estimation needs experience. In this respect, the proposed model is the same as the hydrothermal model [7,20]. However, genome-scale metabolic network models have seen a rapid development over the past few decades to quantify the response of germination to environmental cues at the molecular scales [66,67]. As mechanisms underlying the germination speed and its associated dispersion coefficients are the consequence of the response of metabolic reactions at each physiological stage to environmental variations, the proposed model might open an avenue for linking germination traits to genome-scale metabolic networks.

## 5. Conclusions

We proposed a new conceptual model for seed germination by viewing the germination process as a moving event in a physiological dimension, with the impact of fluctuations of environmental cues and seed heterogeneity represented by an average germination speed and a germination dispersion coefficient. The two model parameters describe the collective impact of soil water, temperature and bioavailable O_2_ on seed germination. To test the model, we conducted field experiments to measure germination and the subsequent seedling development of winter wheat seeds drilled at different dates (to generate a temperature gradient) in plots with different soil water content (to generate a soil moisture gradient). The results show the model accurately reproduces the germination patterns and seedling tillering patterns observed in all treatments. We also assessed the predictive capability of the model by comparing it with data measured on the same site over a period spanning seven years and compared it with the hydrothermal-time model to analyse germination patterns of wheat seeds in different soils measured in pressure chambers. The novelty of introducing a physiological dimension is that it allows seed germination and the subsequent seedling development to be modelled as a seamless continuous physiological process, providing a deeper insight into plant growth dynamics.

## Data Availability

Dataset and a MATLAB code used to create the figures are available at Figshare [68].
